# Mitigation of indomethacin-induced gastrointestinal damages in *fat*-1 transgenic mice via gate-keeper action of ω-3-polyunsaturated fatty acids

**DOI:** 10.1038/srep33992

**Published:** 2016-09-23

**Authors:** Young-Min Han, Jong-Min Park, Jing X. Kang, Ji-Young Cha, Ho-Jae Lee, Migeyong Jeong, Eun-Jin Go, Ki Baik Hahm

**Affiliations:** 1 CHA Cancer Prevention Research Center, CHA Bio Complex, Seongnam, Korea; 2 Laboratory for Lipid Medicine and Technology, Massachusetts General Hospital, Harvard Medical School, Boston, MA 02129, USA; 3 Lee Gil Ya Diabetes and Cancer Institute, Gachon University, Incheon, Korea; 4 Digestive Disease Center, CHA Bundang Medical Center, CHA University, Seongnam, Korea

## Abstract

Non-steroidal anti-inflammatory drugs (NSAIDs) damage the gastrointestinal (GI) epithelial cell membranes by inducing several signals through lipid raft organization after membrane incorporation, whereas ω-3 polyunsaturated fatty acids (PUFAs) relieve inflammation, reduce oxidative stress, and provide cytoprotection, consequent to lipid raft disorganization. Therefore, we hypothesized that ω-3 PUFAs can protect the GI from NSAID-induced damages by initiating the gatekeeper action of cell membranes, subsequent to anti-inflammatory and anti-oxidative actions. Administration of indomethacin (IND) leads to the formation of lipid rafts and activation of caveolin-1; however, no such observations were made upon co-administration of eicosapentaenoic acid (EPA) and IND. In addition, the EPA-induced lipid raft disorganization, caveolin-1 inactivation, and cellular cytotoxicity were inhibited when target cells were knocked-out using G-protein coupled receptor 120 (GPR 120). EPA significantly attenuated IND-induced oxidative damage and apoptosis. IND administration induced significant ulceration, bleeding, and oedema in the stomach or small intestine of wild-type (WT) mice; however, such severe damages to the GI significantly decreased in *fat*-1 transgenic (TG) mice (*P* < 0.001), which exhibited decreased cyclooxygenase-2 expression and apoptosis, decreased interleukin-1β and FAS concentrations, and increased heme oxygenase-1 concentration. Our study indicates that the gatekeeper function of ω-3 PUFAs improves GI safety when administered with NSAID.

The mechanisms by which non-steroidal anti-inflammatory drugs (NSAIDs) induce damage to the gastrointestinal (GI) tract are diverse; however, the primary mechanisms are the inhibition of cyclooxygenase (COX) and subsequent depletion of mucoprotective prostaglandins (PGs)^1^,^2^. Although selective COX-2 inhibitors (*coxib*) or combinations with synthetic PGs or proton pump inhibitors (PPI) were found to be superior to the conventional NSAIDs in reducing side effects, they still await further improvement.

NSAIDs have the ability to diffuse into both synthetic and biological membranes; interact with membrane phospholipid molecules; and considerably modify the membrane’s hydrophobicity such as fluidity, bending stiffness, and permeability, which can alter the ‘gatekeeping functions’ of cells, leading to an inevitable GI mucosal damage such as erosions, ulcers, and bleeding, consequent to membrane pore formation^3^,^4^,^5^,^6^. Therefore, by preventing the changes associated with lipid raft organization, the resulting NSAID-induced GI damage can be minimized. While NSAIDs alter the membrane stability, induce the formation of unstable pores, and cause back-diffusion of luminal acid after membrane rupture, ω-3 polyunsaturated fatty acid (ω-3 PUFAs) can establish liaisons with membranes to strengthen their gatekeeping function and therefore reduce these effects.

The ω-3 PUFAs have already been reported to stabilize the membrane by modifying membrane structure, disturbing membrane lipid raft organization, and inactivating cell signalling relevant to apoptosis^7^. Therefore, we hypothesized that ω-3 PUFAs act as gatekeepers against the cytotoxic effects of NSAIDs. Several reports have already demonstrated the beneficial effects of dietary ω-3 PUFAs against gastric damage induced by ethanol-8,9, aspirin-10,11, NSAIDs-12, and water immersion-restraint stress^13^. However, the results were inconsistent and unconvincing because dietary administration of ω-3 PUFAs led to inconsistent effects, and ω-3 PUFAs were used only as a supplement to other mediators.

To overcome the limitations of the previous studies, we documented the efficacy of endogenously synthesized ω-3 PUFAs against indomethacin (IND)-associated GI damages in *fat*-1 transgenic (TG) mice and their detailed *in vitro* mechanisms, assuming that the gatekeeping action of ω-3 PUFAs can improve GI safety. We compared *fat*-1 TG mice, engineered to carry the *fat*-1 gene encoding n-3 desaturase from the worm *Caenorhabditis elegans,* which is capable of converting n-6 PUFAs to n-3 PUFAs^14^, to wild-type mice. Investigations are being conducted to develop safer NSAIDs such as nitric oxide (NO)-releasing NSAIDs^15^, phosphatidylcholine (PC)-conjugated NSAIDs^16^, and hydrogen sulfide (H_2_S)-releasing NSAIDs^17^, and our study provides information towards the development of ω-3 PUFAs-conjugated NSAIDs, which may enable improved gate-keeping function and, hence, the GI safety.

## Results

### Eicosapentaenoic acid (EPA) acts as a gatekeeper to block IND-induced lipid raft organization and the inactivation of caveolin-1, after binding to G-protein coupled receptor 120 (GPR 120)

**Figure 1:**
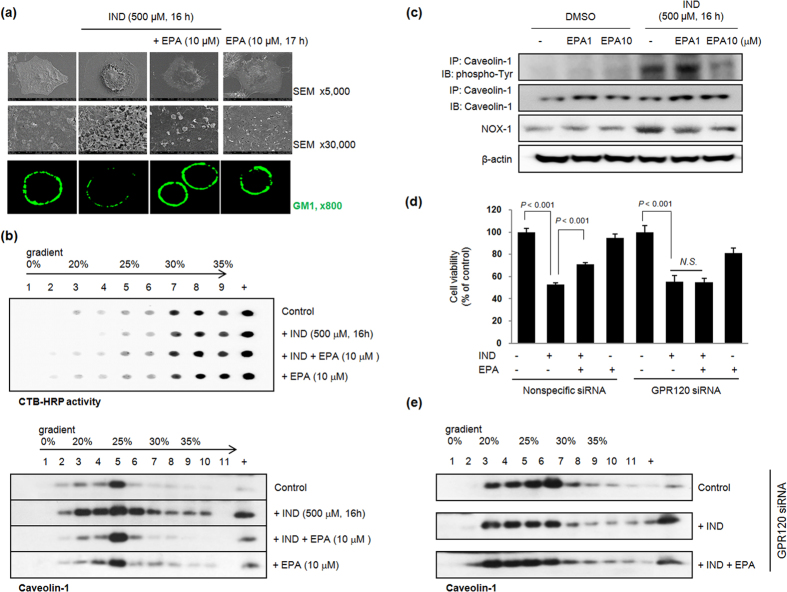
Inhibitory effect of EPA on lipid rafts organization in gastric epithelial cells after 500 μM IND administration (**a**) SEM (upper and middle) and confocal analysis of GM1 (lower) were done in RGM-1 cells, non-transformed gastric mucosal cells pretreated with EPA (10 μM) for 1 hr before 16 h of 500 μM IND administration. Confocal microscopy analysis was done with GM1 staining in RGM-1 cells and their localization of GM1 is seen as green fluorescence via FITC-conjugated GM1 (Magnification at x 800). (**b**) Dot-blot analyses and Western blot for lipid raft fractionated by sucrose density gradient centrifugation RGM-1 cells were treated with vehicle alone (Control) or with 500 μM IND and with 10 μM EPA or not as indicated. The distribution of GM1 in the gradient fractions was determined using 2 μg of protein of each fraction and HRP-CTB. (**c**) Immunoprecipitation was done with caveolin-1 antibody from each homogenate and Western blotting was done with phosphorylated-tyrosin and caveolin-1, respectively. Also western blot with NOX-1 was done. Full length blots are presented in Supplementary Figure 1a. (**d**) Cell survival by MTT assay between ns siRNA-transfected and GPR120 siRNA-transfected RGM-1 cells. In RGM-1 cells transfected with either ns siRNA or GPR120 siRNA, MTT assay was done under the challenge with 10 μM concentration of EPA for 1 hr before IND-treatment (500 μM, 16 h). (**e**) Western blotting for caveolin-1 in fractions obtained by sucrose density gradient centrifugation RGM-1 cells were transfected with GPR120 siRNA and RGM-1 cells were treated with vehicle alone (Control) or with 500 μM IND and with 10 μM EPA or not as indicated. Each fraction was resolved on SDS-PAGE gels and Western blotting using caveolin-1 antibody.

The NSAID-induced perturbation of the membrane structure led to changes in surface tension, membrane partitions, and lipid packing shapes, and altered the stability of the membrane bi-layer, leading to the formation of unstable pores^18^. The partitioning of aspirin or NSAID molecules can further reduce the membrane thickness and decrease the bending stiffness, thus inducing membrane pore formation^18^. To validate the direct cytotoxicity of NSAIDs^18^,^19^, scanning electron microscopy (SEM), which is a type of electron microscope that produces images of a sample by scanning it with focused beam of electrons, was performed 16 hr after IND administration and Fig. 1a shows that IND (500 μM, 16 hr) exposure led to overt cell damage such as cell surface blebs, membrane pore formation, and basement membrane detachment. However, co-administration of EPA and IND significantly mitigated these changes, indicating that EPA exerted gate-keeping functions against IND. Figure. 1a shows cross-linked monosialotetrahexosylganglioside (GM1) lipid micro-domains on the surface of RGM-1 cells; the domains are visualized with cholera toxin subunit B (CTB)-FITC. Since the glycosphingolipid, GM1 ganglioside, was found to partition exclusively within lipid rafts and caveolae, and binding of CTB to ganglioside GM1 is accepted as a marker of lipid rafts, we compared the diffusion patterns among groups. When compared with cells treated with 500 μM IND, which induces microdomain organization, the lipid microdomain clustering induced by cholera toxin cross-linking was significantly diminished in the presence of 10 μM EPA compared to non-treated cells. IND induced the clustering of lipid rafts to form lipid raft organization, whereas co-treatment of IND and EPA mitigated this effect, leading to lipid raft disturbance. Thus, EPA-induced gatekeeping functions contributed to significant protection from immediate NSAID damage. GM1 stainings were further evaluated with GM1 dot-blot analysis to reflect CTB-HRP activity (Fig. 1b). To determine whether IND also affected caveolin-1 localization at lipid raft microdomains, Triton X-100 lysates of RGM-1 cells, which had been treated with IND alone or co-treated with IND and 10 μM EPA, were fractionated on sucrose density gradients. Using serial fractions from gradient centrifugation, caveolin-1, frequently used as lipid raft markers, was strongly accumulated in fractions 3–5 in control cells, whereas organization was noted in IND-treated cells (Fig. 1b). These experiments consistently showed IND-induced lipid raft organization, whereas EPA significantly inhibited IND-induced lipid raft organization. Functionally, caveolin-1, a substrate for non-receptor tyrosine kinases including *Fyn, Abl*, and *Src*, acts as a scaffolding protein and can be phosphorylated on tyrosine 14 by these kinases in response to external stimuli such as reactive oxygen species (ROS). Such tyrosine phosphorylation activates the downstream signalling targets and thus serves as a crucial step for intracellular signalling leading to cytotoxicity^20^. Therefore, we performed immunoprecipitation tests with caveolin-1 after administration of IND alone or in combination with EPA, and Western blotting with phosphorylated tyrosine antibodies. As seen in Fig. 1c, EPA significantly inactivated IND-induced caveolin phosphorylation as well as NADPH oxidase (NOX)-1 expression, signifying that expression of NOX-1, a membrane-bound enzyme complex, increased after IND treatment (Fig. 1c) and is a part of the lipid raft disorganization in membrane caveolae. Furthermore, since IND organizes lipid rafts to send signals that leads to cell death and inflammation, ω-3 PUFAs can block this IND-induced raft organization in order to hinder cell death signals as well as inflammation propagation. GPR 120 is a ω-3 PUFA receptor that mediates various functions such as potent anti-inflammatory and insulin-sensitizing effects^21^. Therefore, in order to determine whether these lipid raft disorganizations were driven by EPA, we repeated the experiments with cells harbouring wild-type GPR120 and GPR120-knockout cells. After RGM-1 cells were transfected with nonspecific and specific GPR120-siRNA, no significant changes were observed in cell growth because GPR120-knockout cells grew normally and their proliferation over 24 hr was not affected by GPR-120-siRNA. GPR120-knockout cells lost the protective effects of EPA (Fig. 1d), suggesting that the action of EPA operates through EPA-bound GPR120. The typical lipid raft disorganization noted with EPA was not seen in GPR120-siRNA cells, despite the administration of EPA (Fig. 1e), signifying that lipid raft disorganization after EPA afforded significant protection from IND-induced cytotoxicity. In conclusion, EPA has gatekeeping action against IND cytotoxicity.

### Gatekeeper action of EPA mitigates apoptosis and oxidative stress

**Figure 2:**
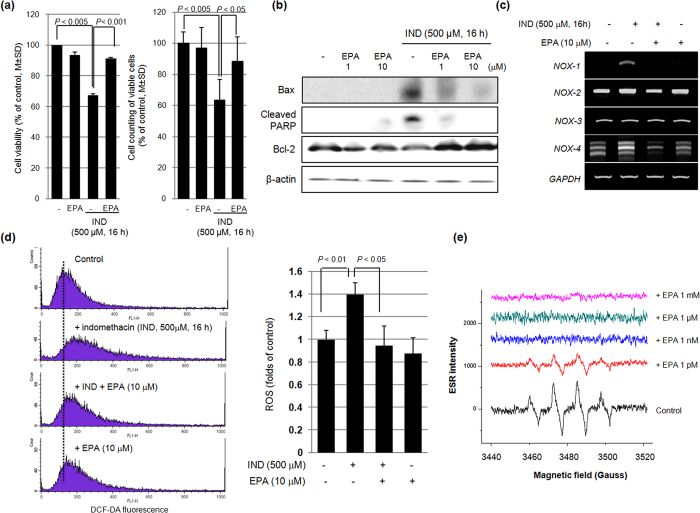
Changes of NOX, apoptosis, and oxidative stress according to IND and combination of IND and EPA (**a**) Cell survival by MTT assay and Cell counting. They were done in RGM-1 cells under the challenge with 10 μM EPA for 1 hr before IND treatment (500 μM, 16 h). (**b**) Western blot for Bcl-2, Bax, and PARP RGM-1 cells were pretreated with EPA (10 μM) for 1 hr and stimulated with IND for 16 hr. Expression of apoptosis mediator was analyzed by immunoblotting. All experiments were done in triplicate. Mean ± SE was calculated from independent experiments. Full length blots are presented in Supplementary Figure 1(b–d). (**c**) RT-PCR for NOXs family RGM-1 cells were pretreated with EPA (10 μM) for 1 hr and stimulated with IND for 16 hr. Expression of NOX family was analyzed by RT-PCR. (**d**) FACS analysis of DCF-DA Cells were treated 10 μM of EPA for 1 hr before IND treatment (500 μM, 16 h) then incubated with fluorescent probe, H_2_DCFDA for 30 min. (**e**) ESR measurement ESR spectra of the DMPO‐OH adducts arising in the Fenton reaction. After administration of EPA, the signal intensity of ESR spectra was decreased in a dose-dependent manner.

Because our previous report has shown that IND was responsible for the production of ROS, which provoked either apoptosis or oxidative stress in the stomach^22^, we hypothesized that the gatekeeping action of ω-3 PUFAs can scavenge ROS and rescue target cells from consequent apoptosis. Since IND-induced cytotoxicity causes oxidative stress^23^, we measured cell viability with an MTT assay after administration of 500 μM IND and found significant decreases in cell viability (Fig. 2a); we also measured the expression of apoptosis-related genes such as Bcl-2, Bax, and PARP after administration of IND alone or in combination with EPA (Fig. 2b). IND (500 μM) significantly increased Bax expression and the cleavage of PARP in RGM-1 cells. However, RGM-1 cells co-treated with IND and 10 μM EPA showed a significant decrease in the expression of Bax and cleavage of PARP (*P* < 0.01). In addition, 10 μM EPA significantly increased the expression of Bcl-2, an anti-apoptotic protein. Since the source of oxidant generations by IND is mostly NOX-1, a tightly controlled multi-component enzyme composed of a membrane-associated catalytic moiety and cytosolic regulatory components, we analysed the changes in the NOX subunits (*Nox-1, Nox-2, Nox-3,* and *Nox-4* mRNA) after administration of IND alone, IND in the presence of EPA, and EPA alone. As shown in Fig. 2c, EPA inhibited the expression of the *Nox1* mRNA (Fig. 2c). We also analysed whether EPA can reduce IND-associated ROS generation in non-transformed gastric mucosal cells, RGM-1 cells (Fig. 2d), with flow cytometric analyses using Dischlorofluorescin diacetate (DCF-DA)fluorescence. After treatment of RGM-1 cells with IND, DCF-DA fluorescence increased; however, co-administration of 10 μM EPA and 500 μM IND led to a significant inhibition of the DCF-DA fluorescence. Electron spin resonance (ESR) using DMPO-free radical adduction is one of the best methods of measuring hydroxyl radicals through spectroscopy (Fig. 2e). ESR measurement using DMPO as an adductor showed significant peaks for hydroxyl radicals generated by the Fenton reaction; however, concentrations of EPA above 1 nM showed significant scavenging of hydroxyl radicals, as depicted by the DMPO-adduct.

### Effect of ω-3 PUFAs on IND-induced gastric damage

**Figure 3:**
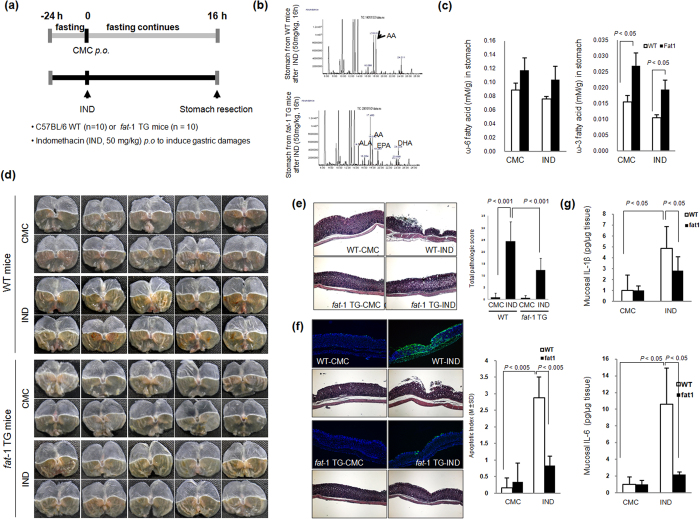
Mitigating effects of ω-3 PUFAs in IND-induced gastric damage (**a**) Protocol for IND-induced gastric damages. The schematic overview: the experimental protocol for IND-induced gastric ulcer using WT and *fat*-1 TG mice. The experimental animal (n = 10) were divided 4 groups, WT with CMC, WT with IND, *fat*-1 TG with CMC, and *fat*-1 TG with IND. (**b**,**c**) Lipid profiles of WT and *fat-*1 TG mice stomach before and after IND administration. The mice were sacrificed 16 h after treatment with IND (50 mg/kg). Stomach tissue of *fat*-1 TG mice and WT mice was analyzed by LC/MS/MS as described in Materials and Methods. (**d**) Gross gastric lesion according to WT and *fat*-1 TG mice before and after IND (50 mg/kg, po). Gross lesion pictures of representational photo in each group were noted. (**e**) Pathological scores according to group gastric changes were quantified from H&E stained sections. In stomach was stained by H&E staining and the pathologic scores were represented as mean ± SD of 10 animals. Statistical significance with the controls was analyzed by one-way ANOVA. (**f**) TUNEL assay for detection of apoptosis. Stomach tissue slides stained with TUNEL staining to detect the apoptotic positivity cells. Graph shows the number of TUNEL-positive cells per high power field (magnification × 400) from at least 10 fields. Each value represents the mean ± SD for 10 mice. (**g**) ELISA assay for IL-1β (upper) and IL-6 (lower). The mucosal cytokine levels of IL-1β and IL-6 were assayed by ELISA method, respectively. All experiments were done in triplicate. Each value represents the mean ± SD for 10 mice.

**Figure 4:**
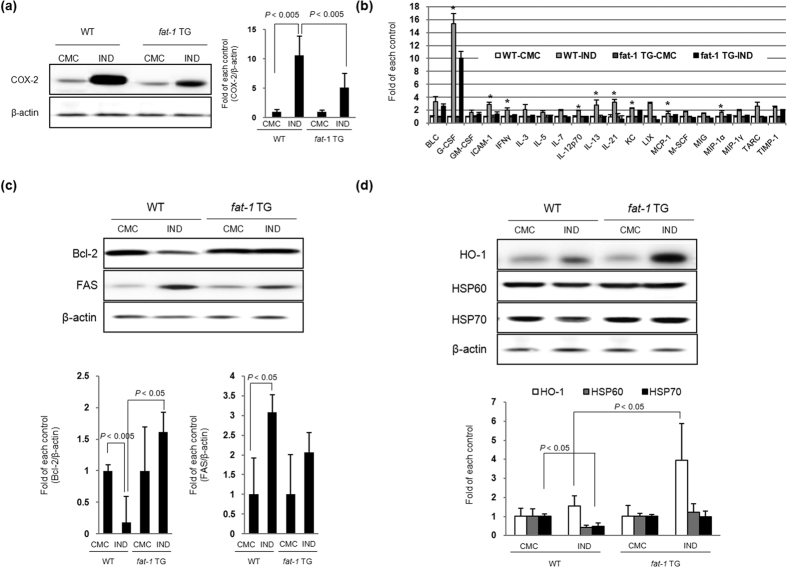
Molecular mechanisms to explain mitigating effects of ω-3 PUFAs in IND-induced gastric damage (**a**) Changes of COX-2 according to group Equal amounts of total protein extracted from stomach tissues were subjected to Western blotting using COX-2 antibody. (**b**) Cytokine array for inflammatory mediators. Equal amounts of total protein extracted from stomach tissues were subjected to cytokine array for inflammatory mediators. The relative intensity of each cytokine was showed (*denoted *P* < 0.01; *versus* WT-CMC). (**c**) Changes of apoptosis mediators according to group. Equal amounts of total protein extracted from stomach tissues were subjected to Western blotting using Bcl-2 and FAS antibodies. (**d**) Changes of HO-1 and HSPs according to group. Equal amounts of total protein extracted from stomach tissues were subjected to Western blot analysis using HO-1, HSP60, and HSP70 antibodies.

To assess the efficacy of ω-3 PUFAs in preventing IND-induced gastric mucosal damage, we induced IND-induced gastric damage in the C57BL/6 wild-type (WT) and *fat*-1 TG mice (Fig. 3a). To prove whether *fat*-1 TG mice can generate ω-3 PUFAs in the stomach, after feeding the rats a normal diet containing ω-6 PUFAs but deficient in ω-3 PUFAs, we measured the profiles of lipid fatty acids in stomach tissue of WT and *fat*-1 TG mice by using GC/MS analysis after administration of IND. As seen in Fig. 3b, arachidonate peaks, representative of ω-6 fatty acids, were detected in both WT and *fat*-1 TG mice, but high levels of α-linolenic acid (ALA), docosa hexaenoic acid (DHA), and EPA were only detected in the stomach of the *fat*-1 TG mice. After measuring all the levels of fatty acids in WT and *fat-*1 TG mice, ω-3 fatty acids levels in *fat-*1 TG mice were found to be significantly higher than those in WT mice (Fig. 3c). Administration of 50 mg/kg of IND led to gastric damage in both the WT mice and CMC-treated WT mice groups, which presented definite gastric ulcer accompanied with brisk gastric haemorrhages (Fig. 3d); however, no significant gross lesions were noted in *fat*-1 TG mice group, which showed only mild erythematous and small sized erosions. Microscopically, exposure to IND for 16 hr induced definite gastric mucosal injury such as ulcers, erosions, and inflamed gastric mucosa. The total pathologic score (Fig. 3e) increased in IND-induced WT groups, but significantly decreased in the IND-treated *fat*-1 TG group. To assess whether the increased gastric mucosal erosive changes were related to apoptotic cell death, we performed a Terminal deoxynucleotidyl transferase-mediated dUTP nick end labeling (TUNEL) assay (Fig. 3f). The number of TUNEL-positive epithelial cells was counted in each of 10 sections and expressed as a percentage of the total epithelial cells. An increase in the number of TUNEL-positive cells was observed in the gastric mucosa of WT mice after IND treatment, but the number was lower in *fat-*1 TG mice. Further, we conducted an ELISA assay using tissue samples to identify whether the rescuing effects of ω-3 PUFAs against IND-induced gastric damage were related to the suppression of interleukin (IL)-1β□ and IL-6, which are known to participate in IND-induced gastric damage. As shown in Fig. 3g, levels of IL-1β and IL-6 in tissues after IND administration were elevated in all groups, except in *fat-*1 TG mice, which showed lowered levels. Although IND imposes its anti-inflammatory action through the inactivation of the COX enzyme, increased COX-2 expression is a representative pro-inflammatory mediator for GI damage^24^. As seen in Fig. 4a, IND administration upregulated the expression of the COX-2 protein in WT mice, but COX-2 expression decreased in *fat*-1 TG mice even after IND administration. To clarify the core proteomes involved in either IND-induced gastric damage or the preventative effects of ω-3 PUFAs in *fat*-1 TG mice, we performed a cytokine antibody array using homogenated proteins isolated from the stomach of WT and *fat*-1 TG mice (Fig. 4b). After IND treatment, the expression of the following genes was significantly upregulated: B lymphocyte chemoattractant (BLC); granulocyte-colony stimulating factor (G-CSF, a neutrophil precursor); GM-CSF; intercellular adhesion molecule-1 (ICAM-1, also known as CD54), interferon gamma (IFN-γ); IL-3, IL-5, IL-7, IL-12, IL-13, and IL-21; keratinocyte chemoattractant (KC); lipopolysaccharide-induced CXC chemokine (LIX); inflammatory protein-1 (MIP-1); macrophage colony-stimulating factor (M-CSF); monokine induced by gamma interferon (MIG, CXCL9); MIP-1α and MIP-1γ; thymus- and activation-regulated chemokine (TARC, CCL17); and the tissue inhibitor of metalloproteinase-1 (TIMP1). However, the expression of these genes decreased in *fat*-1 TG mice (Fig. 4b). Moreover, IND has been demonstrated as an apoptosis-inducing agent in several *in vivo* models^25^. IND induces apoptosis by down-regulating Bcl-2 family members and up-regulating FAS expression. IND treatment in *fat-*1 TG mice inhibited the expression of FAS and induced the expression of Bcl-2 when compared to IND-treated WT mice (Fig. 4c), indicating prevention of gastric damage in *fat*-1 TG mice induced by IND through the inhibition of apoptosis. Hosts can react against oxidative change by activating antioxidant enzyme such as heme oxygenase-1 (HO-1). The HSP family members, as a molecular chaperone, have been suggested to exert their gastro-protective action by protecting the mitochondria and interfering with stress-induced apoptosis. As shown in Fig. 4d, the expression of HO-1 and Heat shock protein (HSP) 70 in *fat*-1 TG mice was higher than in IND-treated WT mice.

### Effect of ω-3 PUFAs on IND-induced intestinal damage

**Figure 5:**
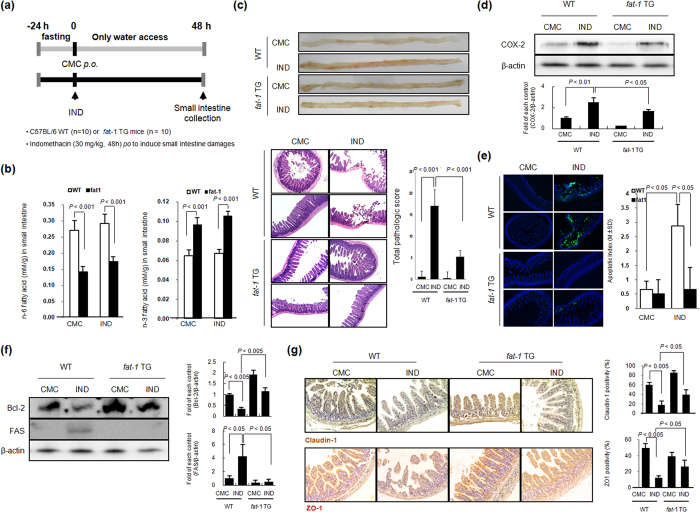
Mitigating effects of ω-3 PUFAs against IND-induced small intestine damage (**a**) Protocol for IND-induced small intestinal damages. The schematic overview: the experimental protocol for IND-induced small intestine injury using WT and *fat*-1 TG mice. The experimental animal (n = 10) were divided 4 groups, WT with CMC, WT with IND, *fat*-1 TG with CMC, and *fat*-1 TG with IND. (**b**) Comparison of concentration of endogenous ω-3 PUFAs between WT and *fat-*1 TG mice. The mice were sacrificed 48 hr after treatment with IND (30 mg/kg, po). Small intestine tissue of *fat*-1 TG mice and WT mice was analyzed by LC/MS/MS as described in Materials and Methods. (**c**) Gross lesion and total pathological score of IND-induced small intestine injury. Gross lesion pictures of representational photo in each group were noted (upper). In small intestine was stained by H&E staining and the pathologic scores were represented as mean ± SD of 10 animals (lower). Statistical significance with the controls was analyzed by one-way ANOVA. (**d**) Changes of COX-2 according to group Equal amounts of total protein extracted from small intestine tissues were subjected to Western blotting using COX-2 antibody. (**e**) TUNEL assay for detection of apoptosis. Small intestine tissue slides stained with TUNEL staining to detect the apoptotic positivity cells. Graph shows the number of TUNEL-positive cells per high power field (Magnification at × 400) from at least 10 fields. Each value represents the mean ± SD for 10 mice. (**f**) Changes of apoptosis mediators according to group Equal amounts of total protein extracted from small intestine tissues were subjected to Western blotting using Bcl-2 and FAS antibodies. Full length blots are presented in Supplementary Figure 2(a–c). (**g**) Changes of Claudin-1 and ZO-1 in small intestine according to group. The expression of Claudin-1 and ZO-1 were investigated by immunohistochemistry. Each value represents the mean ± SD for 10 mice.

IND exhibit toxic effects in the small and large intestine by inducing erosions and ulcers^24^. To assess the clinical efficacy of ω-3 PUFAs on IND-induced intestinal damage, similar to previous gastric damages, we induced damage in the small intestines with IND in WT and *fat*-1 TG mice (Fig. 5a). In order to check the presence of ω-3 PUFAs, ALA, EPA, and DHA in the intestine, we measured the profiles of lipid fatty acids in small intestine tissue of WT and *fat*-1 TG mice before and after IND administration with GC/MS analysis. As seen in Fig. 5b, the levels of ω-3 PUFAs in *fat-*1 TG mice were significantly higher than those in WT mice. However, *fat*-1 TG mice showed significantly decreased levels of ω-6 PUFAs (Fig. 5b). Our model of IND-induced small intestinal damage exhibited linear ulceration with some bleeding and erythematous and oedematous intestinal mucosa extending along the mesenteric border of the jejunum and ileum (Fig. 5c). We noted significant increase in either the gross lesion scores or pathological scores in WT mice administered with IND, but lowered scores in *fat*-1 TG mice. When we compared the experimental groups using tissue samples to identify whether the preventive effects of ω-3 PUFAs against IND-induced small intestinal damage were related to the suppression of inflammation and apoptosis, treatment with IND was found to markedly induce the expression of COX-2, but the expression of COX-2 in *fat*-1 TG mice after IND-treatment was lower than that in the IND-treated WT mice (Fig. 5d). To assess apoptotic cell death in the small intestine, we conducted a TUNEL assay (Fig. 5e). Increased TUNEL-positive cells were observed in the small intestinal mucosa after IND treatment in WT mice, but the numbers of TUNEL-positive cell were significantly lower in *fat*-1 TG mice after IND-treatment. IND induces apoptosis by down-regulating Bcl-2 family members and up-regulating FAS expression (Fig. 5f), and these levels in *fat*-1 TG mice were changed. Weakness in tight junctions is mainly involved in the pathogenesis of NSAID-induced enteropathy, and as such, we measured changes in the tight junction proteins, claudin-1 and Zonula occludens (ZO)-1, in each group^26^. Tight junctions are also very important factors in cell structures when considering NSAID toxicity. Studies have shown a decrease in key tight junction proteins such as ZO-1 and claudin-1 correspond to an increase in intestinal permeability and decrease in transepithelial resistance^26^. As seen in Fig. 5g, the abundance of claudin-1 and ZO-1 in the small intestinal mucosa is shown in either WT or *fat*-1 TG mice before IND administration. However, the levels of claudin-1 and ZO-1 significantly decreased after IND administration in WT mice, but were unalteredin *fat*-1 TG mice. All of these *in vivo* animal model studies consistently suggested that ω-3 PUFAs exert significant rescuing actions against IND-induced gastric or intestinal damages through the orchestration of potent anti-inflammatory, anti-apoptotic, and epithelial homeostatic actions.

### Comparison of ω-6/ω-3 PUFAs in the stomach and small intestine between *fat*-1 TG mice and mice administered with exogenous ω-3 PUFAs

The above *in vivo* data support the fact that ω-3 PUFAs can significantly mitigate NSAID-induced GI damages. Based on this, we have raised two queries: (i) how much exogenous ω-3 PUFAs would be required to ameliorate NSAID-induced GI damage, and (ii) what is the mode of action to explain the membrane changes due to NSAIDs administration. In our experiments, different doses of ω-3 PUFAs were administered to WT mice and the n ω-6/ω-3 PUFA ratio was measured in the stomach and small intestine and compared the levels to those in *fat*-1 TG mice. There were significant differences in the ω-6/ω-3 PUFAs ratio between WT and *fat*-1 TG mice in both the stomach and small intestine. In order to match the similar ameliorating effect on IND-induced gastric damage, 3 g/60 kg dosing of exogenous ω-3 PUFAs was administered (data not shown). For IND-induced small intestinal damage, 1 g/60 kg dosing of exogenous ω-3 PUFAs should be considered^27^. Conclusively, the gatekeeper function of ω-3 PUFAs seems to be very critical in determining the NSAID-associated cytotoxicity, such as lipid raft organization and NOX-1 activation through caveolin-1 phosphorylation of NSAIDs. Based on this *in vivo* data, ω-3 PUFAs-conjugated NSAIDs or a combination of ω-3 PUFAs and NSAIDs may mitigate NSAID-induced GI damage; however, a detailed clinical trial is still required to verify this effect.

## Discussion

**Figure 6:**
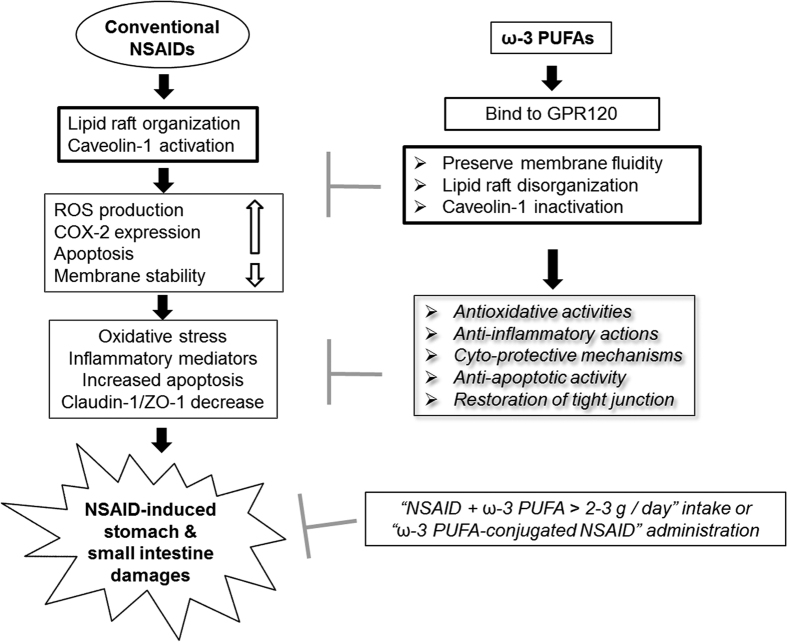
Summary explaining the mitigating action of ω-3 PUFAs against NSAIDs-induced GI damage. As a proposed pathway for ω-3 PUFAs-mediated inhibition of IND-induced GI damages, our study significantly opens the possibility of ω-3 PUFAs conjugated NSAID or the combination of ω-3 PUFAs and NSAID as potential GI-safer NSAIDs.

In the current study, we have elucidated whether ω-3 PUFA-conjugated NSAIDs or a combination strategy of NSAIDs and ω-3 PUFAs are capable of mitigating NSAID-induced GI damage, as well as lipid raft microdomain disturbance accompanied with significant caveolin-1 inactivation and NOX-1 inhibition, by using ω-3 PUFAs (fig. 6). Our findings were inconsistent with previous publications that studied exogenous ω-3 PUFAs administration, as we demonstrated that administration of greater than 3 g/60 kg of ω-3 PUFAs resulted in lipid profiles as seen in *fat*-1 TG mice, and exhibited protective effects against NSAID-induced GI damage. Conclusively, we anticipate that ω-3 PUFAs-conjugated NSAIDs could be used to improve GI safety upon NSAID administration, and exceed the efficacies of current *coxib* or other conjugated NSAIDs such as NO-releasing NSAID, H_2_S-releasing NSAIDs, and PC-conjugated NSAIDs. Since conventional NSAIDs, not *coxib*, provide additional effects of neuro- or CV-protection, despite the adverse effects on the GI, and because PPIs were proven to exaggerate NSAID-induced enteropathy^28^, our results are important with regards to mitigating NSAID-associated GI damage.

NSAIDs are the most frequently used drugs worldwide to control pain or inflammation. However, they are known to cause GI damage, from mild erosive changes to serious ulceration, which may lead to fatal complications including bleeding and perforation of stomach in the elderly; further, administration of *coxib*s leads to decreased GI injuries, but GI-safe *coxib*s also carry cardiovascular (CV) risks dependent on COX-1 expression^2^,^29^. Although c*oxib*s were proven superior in reducing GI risk than non-selective NSAIDs alone or combination with PPI, renal complications and CV risks are still common problems associated with these drugs.

Dietary ω-3 PUFAs were reported to be beneficial in the treatment of several types of GI disease models including ethanol-induced gastric damages, pylorus ligation, cold-restrain or water-immersion stress in rats, aspirin or IND-induced gastric damages, and even cancer prevention^8^,^9^,^10^,^11^,^12^,^13^,^30^. However, these studies had insufficient evaluations of efficacy, obscure underlying gastro-protective mechanisms, and limited execution through administration of fish oils or other limited sources of fatty acid (FA). Besides GI diseases, considerable research has been conducted to evaluate the potential therapeutic effects of fish oils, an excellent source of long-chain ω-3 PUFAs such as EPA and DHA, in numerous conditions including arthritis, coronary artery disease, inflammatory bowel disease, bronchial asthma, neural injury, ocular disease, sepsis, and cancer. Additional investigations on the use of supplementation with fish oils in patients with neural injury, cancer, ocular diseases, and critical illness have recently been conducted^31^. In these investigations, ω-3 PUFAs in fish oils reduced the production of inflammatory cytokines such as Tumor necrosis factor (TNF)-α, IL-6, and IL-1β^32^. In the current study, we found that ω-3 PUFAs synthesized in *fat*-1 TG mice were effective against either IND-induced gastric damage or small intestinal damage, with similar anti-inflammatory and anti-apoptotic mechanisms. Furthermore, they inhibited IND-induced chemokines and other types of cytokines, but increased HO-1 concentration upon gastric damage and strengthened the tight junction, claudin-1 and ZO-1 resulted from the damage. Considering of the potential clinical applications, we measured the optimal concentrations of ω-3 PUFAs accumulated within organs with dietary administration of ω-3 PUFAs in our previous study^27^.

In order to improve GI safety, NO-releasing NSAID, H_2_S-releasing NSAIDs, and PC-conjugated NSAIDs are under active investigation or clinical trials^16^,^17^,^18^,^33^. However, NSAID-induced GI damage goes beyond authentic COX inhibition, including changes to the hydrophobicity, fluidity, biomechanics, and permeability of extracellular and membrane phospholipids^6^. Thus, substances that stabilize these changes in the NSAID-contacted membrane, inhibit the formation of unstable pores, prevent the back-diffusion of luminal acid, and prevent membrane rupture are important candidates to consider. We hypothesized ω-3 PUFAs to be good candidates because membrane phospholipids containing ω-3 PUFA acyl chains modify the structure and composition of membrane rafts, affect cell signaling to prevent cytotoxic changes, and maintain the membrane fluidity, phase behavior, permeability, fusion, and resident membrane protein activity^34^.

Although the structure, size, and functionality of lipid rafts have been determined, nano-scale domains in the plasma membrane formed through favourable lipid–lipid and lipid–protein interactions^35^ are still under investigation. The significance of membrane micro-domains in health and disease studies is growing because lipid rafts can be potentially modified by one’s diet, particularly by dietary FAs^36^. Although it is not clear yet whether dietary PUFAs can really be incorporated into raft lipids, the role of lipid rafts in NSAIDs-associated apoptosis and inflammation is to disturb lipid-raft-associated negative signaling after NSAID administration. In order to explore these mechanisms^7^, using solid-state Nuclear magnetic resonance spectroscopy, one study explored the molecular organization of mixtures with sphingo-lipids or cholesterol and found that ω-3 PUFAs clearly disrupted lipid raft domain organization^7^. This is corroborative with the results of our study, wherein significant anti-inflammatory and anti-apoptotic action against NSAID-induced damage was related to the lipid raft disorganizing action of ω-3 PUFAs. Briefly, the processes disrupting the molecular organization of membrane lipid rafts and proteins are as follows: ω-PUFAs are incorporated into the raft, cholesterol is redistributed to non-rafts, rafts are declustered, and non-raft proteins are sequestered into declustered rafts^37^. Lipid rafts behave as major modulators of membrane geometry, rendering lateral movement of molecules, traffic and signal transduction, and NSAIDs-associated pro-apoptotic and apoptotic signaling pathways, which start from the raft area^38^. During this process, since ω-3 PUFA chains reorganize the molecular architecture of plasma membrane sphingo-lipid-cholesterol-enriched lipid rafts, so called ‘disturbed lipid rafting’ with ω-3 PUFAs inhibits the apoptotic signal, mitigates IND-induced cyto-toxicity and can deactivate raft-associated proteins such as death receptor proteins, protein kinases, and apoptosis-associated calcium channels^39^,^40^,^41^.

Whilst not being explored in this current study, lipid raft domains optimize the clustering of signaling proteins at the membrane to facilitate efficient cell signaling, which is required for CD4^+^ T cell activation and differentiation, and ω-3 PUFAs increase the levels of anti-inflammatory lipid mediators, EPA- and DHA-derived lipid mediators, called resolvins^42^. Instead, we showed significant decreases in several kinds of inflammatory mediators. The ω-3 PUFAs were proven to be mediated through GPR120, which is a member of the rhodopsin family of GPRs^21^,^43^,^44^. Among five orphan receptors, GPR 40, GPR41, GPR43, GPR84, and GPR 120 activated by FAs, GPR120 binds to caveolin-1, a family of integral membrane proteins that are the principal components of caveolae membranes. These are a special type of lipid rafts and regulate cell function including signaling platform for GPCRs, certain tyrosine kinase receptor, and these rafts/caveolae can influence redox signaling^45^. In our study, we documented these lipid raft disorganizations after administration of ω-3 PUFAs were mediated through GRP120.

In conclusion, like omega-3-acid ethyl ester capsules (Lovaza, Omarcor), which have already been launched in clinics to prevent atherosclerosis, the introduction of ω-3 PUFA-conjugated NSAIDs or a combination of ω-3 PUFAs and NSAID can improve GI safety with guaranteed reduction in CV risks^33^. Because ω-3 PUFAs have been proven to attenuate cyto-toxicity, inhibit lipid raft organization, relieve oxidative stress, and impose anti-inflammatory effects, ω-3 PUFA-based NSAIDs should be considered for developing next-generation GI-safe NSAIDs.

## Materials and Methods

### Reagents

EPA among ω-3 PUFAs and IND as NSAID were all purchased from Cayman and Sigma Aldrich (St. Louis, MO), respectively. Primers for RT-PCR were synthesized by Macrogen (Seoul, Korea). Antibodies were purchased from Cell Signaling Technology (Beverly, MA) and Santa Cruz Biotechnology (Santa Cruz, CA). Horeseradish peroxidase-conjugated anti-mouse/rabbit/goat IgG was purchased from Santa Cruz Biotechnology.

### Cells and Cytotoxicity assay

The rat gastric mucosal cells, RGM1, were maintained at 37 °C in a humidified atomosphere containing 5% CO_2_ and cultured in Dulbecco’s modified Eagle’s medium containing 10% (*v*/*v*) fetal bovine serum and 100 U/ml penicillin. Cells were treated with IND or EPA in DMSO or Ethanol, respectively, and used at the final concentrations indicated in the text and in figure legends. Cell cytotoxicity was measured by MTT, [3-(4,5-dimethylthiazol-2-yl)-2,5-diphenyltetrazolium bromide], assay.

### DCF-DA measurement

For detect the accumulation of ROS in RGM1 cells was monitored using the fluorescence-generating probe DCF-DA. Cells were rinsed with HBSS solution and loaded with 10 μM DCF-DA. After 30 min incubation at 37 °C cells were analyzed with a flow cytometry.

### ESR spectroscopy for hydroxyl radical measurement

Various concentrations of SAC were added to a total volume of 200 μl containing 0.05 mM FeSO4, 1 mM H_2_O_2_, 1 mM 5,5-dimethylpyrroline-Noxide (DMPO, Sigma), 5-*tert*-Butoxycarbonyl-5-methyl-1-pyrroline-N-oxide(BMPO, Enzo, Plymouth Meeting, PA, USA), and 50 mM, sodium phosphate at pH 7.4 at room temperature. Reactions were initiated by adding H2O2. After incubation for 1 min, aliquots of the reactions were transferred to a quartz cell, and the spectrum of DMPO-OH and BMPO-OH was examined using an ESR spectrophotometer (JES-TE300, JEOL, Tokyo, Japan), under the following conditions: magnetic field, 338.0 ± 5.0 mT; microwave power, 4.95 mW; frequency, 9.421700 GHz; modulation amplitude, 5 mT; sweep time, 0.5 min; and time constant, 0.03 s.

### Isolation of lipid rafts from total cell lysates

RGM1 cells were seeded 2–7 × 10^7^ in the 10 cm cell culture plates and incubated for overnight. After 1 day, we treated with IND or EPA as following groups. And then cells were gathered that lysed with Cell Lysis buffer with Triton X-100. The caveolae/rafts isolation kits were purchased from Sigma (Saint Louis, Missouri, USA) and used according to the manufacturer’s instruction. Representative amounts of each fraction from the gradient were analyzed by immunochemistry and immunoblotting with anti-caveolin1 and Cholera Toxin B Subunit-Peroxidase (CTB-HRP) as caveolae and rafts markers.

### Animals

The C57BL/6 mice were purchased from Orient bio (Seoul, Korea) and *fat*-1 TG mice on a pure C57BL/6 background were provided by co-author, Dr. Kang JX (Harvard Medical School, Boston, MA)^14^, respectively. They were bred and genotyped in our facility for the project. The *fat*-1 TG mice were verified as previously described by PCR using tail DNA extracted. The sequences of PCR primers were recommended by Jackson Laboratories and Dr. Kang JX. Animals were handled in an accredited animal facility in accordance with the Association for Assessment and Accreditation of Laboratory Animal Care International (AALAC in Gachon University) policies. The animal study was approved by Center of Animal Care and Use (CACU) committee in Gachon University (Approve number. LCDI-2010-0038).

### Animal experimental procedure

Five-week-old female C57BL/6 and *fat*-1 TG mice were housed in a cage maintained at 23 °C in a 12 h/12 h light/dark cycle under specific pathogen-free conditions. After 1 week of adaptation, 6-week-old mice weighing 18–22 g were used for the experiments. Gastric ulcers and small intestinal injury were induced in mice by intra-gastric administration of IND. We divided four groups: C57BL/6 WT and *fat*-1 TG mice were fasting for 24 h and the given either saline or IND by oral gavage. For gastric ulcer damage and small intestinal injury model mice were sacrificed 16 and 48 hr later, respectively and then stomach and small intestine tissue were collected to analyze detailed molecular study.

### Hematoxylin and Eosin (H&E) staining and immunohistochemistry

For histopathological analysis, the stomach and small intestine were fixed in 10% neutralized buffered formalin, processing using the standard method and embedded in paraffin. Sections of 4-μm thickness were then stained with H&E. The glandular mucosae of corpus and antrum were examined histologically. Pathologic index was graded according to criteria. Pathological data and slides were blindly reviewed by three independent GI specialists. For the immunohistochemistry, paraffin unstained slides were deparaffinized, rehydrated, and boiled in 100 mM Tris-buffered saline (pH 7.6) with 5% urea in an 850 W microwave oven for 5 min each. Sections were also incubated with Claudin1 and ZO1 antibody in the presence of 0.1% bovine serum albumin and finally incubated for 24 h at 4 °C. The sections were counterstained with hematoxylin.

### TUNEL assay

To determine cytotoxicity using TUNEL method using *in situ* cell apoptosis detection kits (Promega, Madison, WI). The paraffin block tissue slides were depaffinized and permeabilized with paraformaldehye. The slides were incubated with the UNEL reaction mixture contatining TdT and fluorescein-dUTP or TMR-dUTP. During this incubation step, TdT catalyzed the attachment of fluorescein-dUTP, to free 3′OH ends in the DNA and then visualizing the incorporated fluorescein with a fluorescence microscope.

### Cytokine array and ELISA

Mouse cytokine antibody array were purchased from R&D Systems and carried out strictly according to manufacturer’s instruction. Three hundred micrograms of a representative case of each group was used for this assay. ELISA kits for mouse IL1β and IL-6 (R&D Systems) were purchased and used strictly according to the manufacturer’s instruction.

### Statistical analysis

The data are presented as means ± SD. The Tukey test or the Student t for unpaired results was used to evaluate differences between more than 3 groups or between 2 groups, respectively. Differences were considered to be significant for values of *P* < 0.05.

## Additional Information

**How to cite this article**: Han, Y.-M. *et al.* Mitigation of indomethacin-induced gastrointestinal damages in *fat*-1 transgenic mice via gate-keeper action of ω-3-polyunsaturated fatty acids. *Sci. Rep.* **6**, 33992; doi: 10.1038/srep33992 (2016).

## Supplementary Material

Supplementary Information
