# Supplementary material for: Mitigation of indomethacin-induced gastrointestinal damages in fat-1 transgenic mice via gate-keeper action of ω-3-polyunsaturated fatty acids
Source: Sci Rep. 2016 Sep 23;6:33992. doi: 10.1038/srep33992 (PMC5034283; doi:10.1038/srep33992)

***Supplementary information***

**Mitigation of indomethacin-induced gastrointestinal damages in *fat*-1 transgenic mice via gate-keeper action of ω-3-polyunsaturated fatty acids**

***Short Title: Gate-keeper action of* ω-*3-PUFAs prevents indomethacin-induced gastro-enteropathy***

Young-Min Han^1^, Jong-Min Park^1^, Jing X Kang^2^, Ji-Young Cha^3^, Ho-Jae Lee^3^, Migeyong Jeong^1^, Eun-Jin Go^1^, Ki Baik Hahm^1,4^

YMH and JMP are equally contributed first authors.

^1^CHA Cancer Prevention Research Center, CHA Bio Complex, Seongnam, Korea

**^2^**Laboratory for Lipid Medicine and Technology, Massachusetts General Hospital, Harvard Medical School, Boston, MA 02129, USA

^3^ Lee Gil Ya Diabetes and Cancer Institute, Gachon University, Incheon, Korea

^4^Digestive Disease Center, CHA Bundang Medical Center, CHA University, Seongnam, Korea

**Correspondence to: Professor Ki Baik Hahm, MD. Ph.D** CHA Cancer Prevention Research Center, CHA University CHA Bio Complex, 335 Pangyo-ro, Bundang-ku, Seongnam City, Kyunggi-do 463-400, Korea

Tel.: +82 31 881 7178, Fax: +82 31 881 7250 E-mail: [hahmkb@cha.ac.kr](mailto:hahmkb@cha.ac.kr)

**Supplementary Figure 1.**


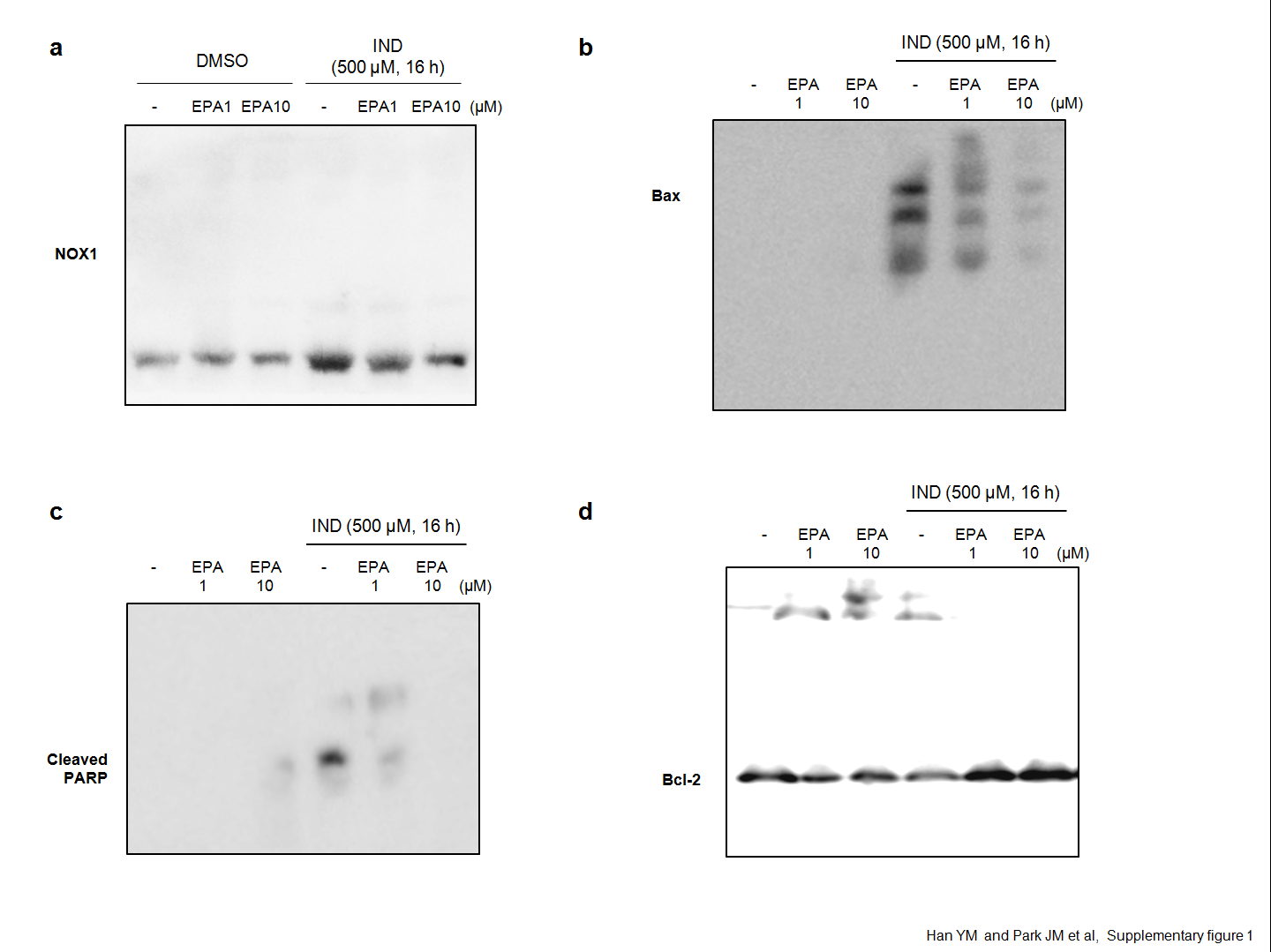


**Supplementary Figure 2.**


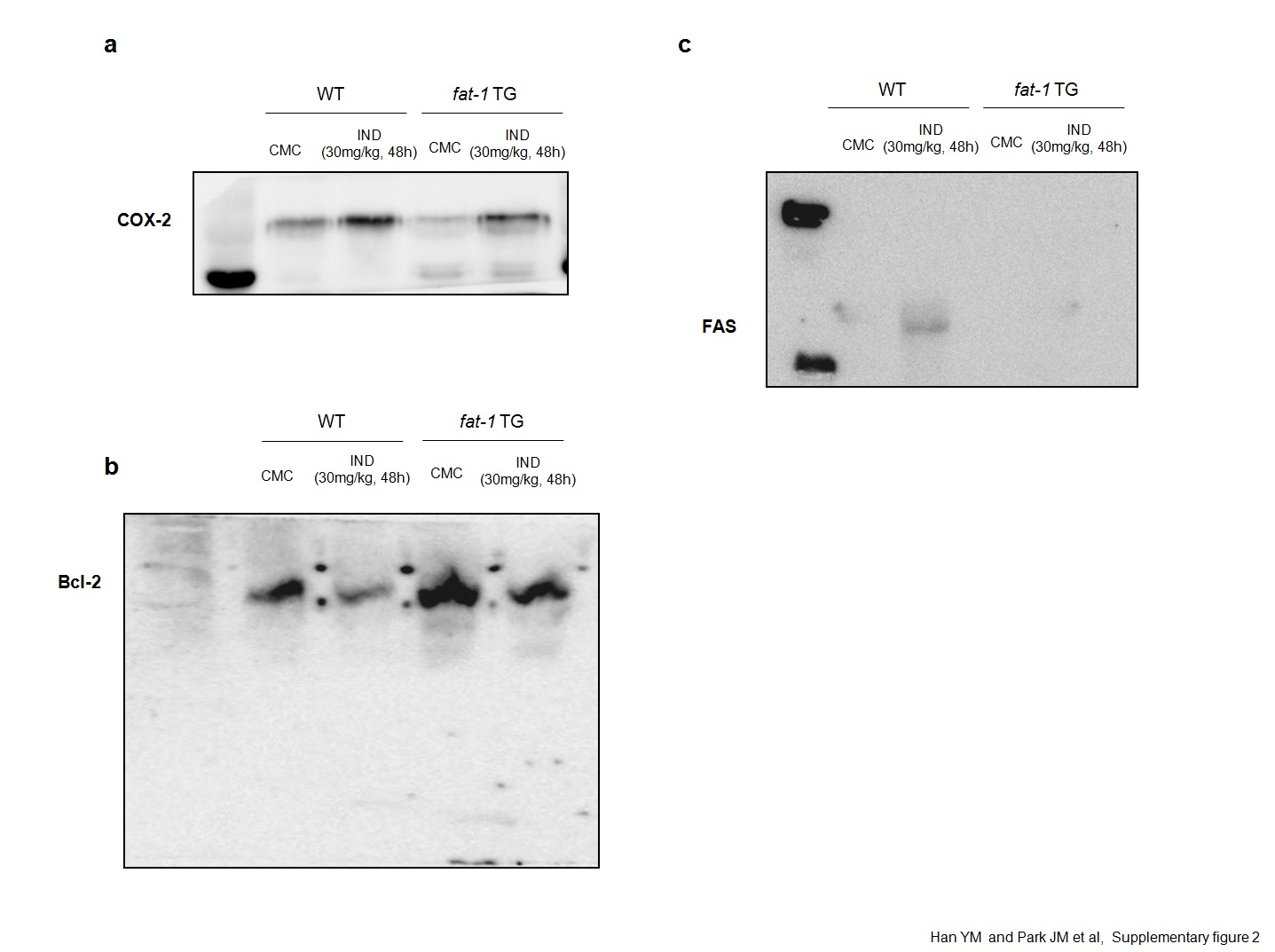

Supplement: Supplementary Information [file srep33992-s1.doc]
